# PLA-ZnO/TiO_2_ Nanocomposite Obtained by Ultrasound-Assisted Melt-Extrusion for Adsorption of Methylene Blue

**DOI:** 10.3390/nano12234248

**Published:** 2022-11-29

**Authors:** Marlene Andrade-Guel, Christian J. Cabello-Alvarado, Gregorio Cadenas-Pliego, Carlos Alberto Ávila-Orta

**Affiliations:** 1 Centro de Investigación en Química Aplicada, Saltillo 25294, Mexico; 2 CONACYT—Centro de Investigación en Química Aplicada, Saltillo 25294, Mexico

**Keywords:** nanocomposite, PLA, ZnO/TiO_2_, adsorption, methylene blue

## Abstract

Access to fresh water is an increasing concern worldwide. The contamination of this vital liquid is largely caused by discharges of pollutants into rivers and seas from different types of industries. Waste dyes from different industries have been classified as harmful to health. In this study, polymeric nanomaterials based on polylactic acid (PLA) and nanoparticles of titanium dioxide (TiO_2_) and zinc oxide (ZnO) modified by ultrasound-assisted extrusion were obtained. These materials were evaluated by FTIR, DRX, TGA, DSC, SEM and methylene blue adsorption. From the results of the physicochemical characterizations, it was possible to observe the presence of TiO_2_ and ZnO nanoparticles dispersed in the polymeric matrix, increasing the crystallinity and thermal stability of the polymer. In addition, a good dispersion of the nanoparticles could be seen by means of SEM, due to the extrusion assisted by ultrasound. The methylene blue dye adsorption tests revealed that the best result was 98% dye adsorption in a time of 13 min for the 1.5% PZT sample. Additionally, this material could be used for 3 adsorption cycles without affecting its adsorbent properties.

## 1. Introduction

Data from the World Health Organization (WHO) mention that around 2400 million people do not have access to basic sanitation facilities, and more than one billion people do not have access to safe drinking water. It is expected that in 2050 the total population will be nine billion people, and many of them will not be able to obtain a supply of this vital liquid [1]. Access to fresh water is limited due to its contamination by waste water discharged by different types of industries such as textile, paper and dyestuff. There are different types of polluting dyes that are considered to affect natural water resources in an important way [2]. The constant presence of different organic dyes in wastewater gives rise to serious environmental and health problems. There are a number of processes for wastewater treatment depending on the desired degree of treatment to be reached. These can be classified into three categories: physical, chemical and biological [3]. Some techniques applied for water sanitation are coagulation, chemical precipitation, ion exchange, reverse osmosis, advanced oxidation and adsorption, among others [4]. Adsorption is a versatile method to remove various contaminants present in wastewater. This process is produced by interactions of chemical ranges through van de Waals forces, which allows particles, molecules or ions to be trapped or retained on the surface of a material [5]. Methylene blue (MB) is a colorant used in different textile and dye industries. It is cationic in nature and particularly resistant to many conventional treatments. For humans, chronic exposure to methylene blue affects different parts of the body. In particular, the kidney is the organ most affected since the accumulation of this chemical causes kidney function failure due to damage to the kidneys [6].

TiO_2_ in its different crystalline phases is a good photocatalyst due to its high refractive index (2.74) and its ability to absorb wavelengths within the UV-Vis range [7]. TiO_2_ is a material that presents interactions between molecular oxygen and the surfaces adjacent to it. This phenomenon plays a key role in many important processes, such as catalytic oxidation reactions, chemical detection and photocatalysis. While oxygen weakly interacts with fully oxidized TiO_2_, it causes an excess of electrons in TiO_2_ samples. Electron excess originates from intrinsic reducing defects (oxygen vacancies and titanium interstitials) and doping, forming polarized states of Ti^3+^ in the band gap near the bottom of the conduction band [8].

Different nanometric materials, due to their size, can be used as adsorbents with great potential for removing chemical compounds. Nanometric-sized TiO_2_ is an excellent option in the purification of water resources, especially for the removal of heavy metals and decontamination of organic compounds. Some studies report that mesoporous nanometric TiO_2_ deposited on cellulose rapidly removes Pb^2+^ from wastewater. It has been reported that the anatase phase of TiO_2_ has a tendency to form hydrogen bonds by interacting with organic contaminants [9]. 

ZnO is widely used as a semiconductor (Eg = 3.37 eV), magnetic material, electroluminescent material, UV-absorber, piezoelectric sensor and actuator, field emission displaying material, thermoelectric material, gas sensor, a constituent of cosmetics, etc. [10]. In addition, it possesses an excellent effective surface area and leads to higher adsorption of organic molecules, while enhanced photocatalytic activities of this material lead to efficient degradation of dyes in water. This type of photocatalyst in wastewater with a high pH level (pH 11.5) has been monitored and has exhibited great stability [11].

ZnO nanoparticles have been studied as potential dye adsorbents. Some authors report that this type of nanoparticle presents selective adsorption of MB, being a good candidate for the elimination of MB in wastewater [12]. One way to increase its adsorption and antimicrobial properties is to modify its surface, that is, to add functional groups to its surface. The surface modification process of nanoparticles consists of the union of different organic or inorganic materials through covalent bonds or non-covalent bonds, such as hydrogen bonds or electrostatic or van der Waals interactions. Incorporating functional groups such as O-H, C-O and COOH has been reported to increase dispersion in polymeric matrices and their interaction with other molecules [13].

TiO_2_ and ZnO can degrade contaminants in the form of colloidal solutions and in particle suspension systems [14,15]; however, the recycling process is complicated and causes secondary pollution [16]. For this reason, preference has been given to the incorporation of TiO_2_ and ZnO in polymeric matrices. For example, TiO_2_ with cellulose has been reported [17], demonstrating the degradation of basic organic compounds (99% methylene blue) and acids (100% acid violet and methyl violet), in polyurethane with the degradation of phenolic contaminants [18]. Polymeric membranes with fluorinated polyvinylidene-ZnO have been studied for the decontamination of water flows [19], as well as the incorporation of these nanoparticles into polyethylene (PE) membranes, retaining 99% of indigo blue [20]. One of the less-studied biodegradable polymeric matrices in water treatment is polylactic acid (PLA). PLA is a thermoplastic polymer derived from lactic acid (LA) that can be used in the manufacturing of textiles. The production of 90% of LA in the global market is by lactic acid bacteria (LAB), which can stereoselectively produce L or D-LA [21]. Finally, the ultrasound-assisted melt-extrusion method (USME) is a high-quality process used to produce polymeric compounds with an adequate homogeneous dispersion [7].

The interest in obtaining polymeric nanocomposites with biodegradable materials for the good of society has increased, due to the growing concern about environmental contamination from synthetic polymers and microplastics [22]. This study reports the production of PLA polymeric nanocomposites and modified TiO_2_ and ZnO nanoparticles by ultrasound-assisted extrusion for the adsorption of methylene blue dye.

## 2. Materials and Methods

### 2.1. Materials

Polylactic acid (PLA) resin Ingeo biopolymer 6260D was provided by NatureWorks with a melt index of 65 g/10 min. To prepare the nanocomposites, TiO_2_ nanoparticles anatase phase (21 nm) and ZnO nanoparticles (<100 nm) were purchased from Sigma Aldrich (Saint Louis, MO, USA). Methyl blue (MB) was obtained from Sigma Aldrich (Saint Louis, MO, USA) and distilled water with a pH of 7 was used as a solvent to obtain the aqueous solutions.

### 2.2. Surface Modification of ZnO/TiO_2_

The surface modification of ZnO/TiO_2_ with lactic acid by ultrasound energy followed the protocol of Andrade-Guel et al. The reaction time for the modification was 90 min [23].

### 2.3. Composite Preparation by an Ultrasound-Assisted Melt-Extrusion Process

PLA-ZnO/TiO_2_ polymer nanocomposite preparation was carried out using an ultrasound-assisted melt-extrusion process with a lab-sized twin-screw extruder from Thermo Scientific (model Prism TSE-24MC) with a screw diameter of 24 mm and L/D ratio of 40:1. This was assisted by a catenoidal ultrasonic tip (Branson Ultrasonics Corp., CT; D, 51.27 cm). The extruder was connected to a homemade ultrasonic generator (15 to 50 kHz, 100% of 750 W). The temperature profile was a flat one, at 190 ± 1 °C in all sections of the extruder, with a screw speed of 80 rpm. As a post-extrusion system, a cooling bath was used at the outlet of the die with a pelletizer (Thermo Fisher, Waltham, MA, USA). Table 1 shows the concentrations of the samples obtained for this study.

### 2.4. Characterization Techniques

#### 2.4.1. Fourier Transform Infrared (FTIR)

A Magna Nicolet 550 spectrometer was used for the FT-IR analysis; 100 scans and a resolution of 16 cm^−1^ were applied in the range of 400 to 4000 cm^−1^. The samples had previously been dried in a vacuum oven at 100 °C for 15 h and were subsequently supported on KBr granules.

#### 2.4.2. X-ray Diffraction

XRD (X-ray diffraction) was carried out to obtain a structural analysis using a Rigaku Smartlab diffractometer operating at 40 kV and 40 mA with a stability of 0.01%/8 h. Measurements for each system were made in the 2θ range from 10° to 80° with a step size of 0.02 and a count rate of 10 s/step. The crystallinity index was calculated by dividing the crystalline area by the total area (crystalline + amorphous).

#### 2.4.3. Thermogravimetric Analysis (TGA)

The thermogravimetric analysis (TGA) was carried out under the ASTM E-1131 standard, using a Q500 thermal analyzer from TA Instruments. The TGA thermal curve of the samples was obtained. The instrumental conditions were as follows: a heating rate of 10 °C/min and a sample mass of 10 mg. Calibration was carried out with calcium oxalate as the standard. The temperature range was 30–700 °C, up to 600 °C under an N_2_ AP (50 mL/min) atmosphere and up to 700 °C under CO_2_ (50 mL/min).

#### 2.4.4. Differential Scanning Calorimetry (DSC) (ASTM D3418)

A Discovery Series 2500 thermal analyzer from TA Instruments, with a DSC cell, was used for the measurements, under the following experimental conditions. Indium was used as the calibration standard. Samples of ca. 9.0 mg were placed in aluminum pans and were subjected to a heating/cooling/heating cycle at a rate of 10 °C/min under an N_2_ high purity (50 mL/min) atmosphere. The temperature range was 0 °C to 220 °C.

#### 2.4.5. Scanning Electron Microscopy (SEM)

The samples were evaluated using a JEOL model JSM-7401F field emission scanning electron microscope with an EDAX EDS detector. The operating conditions in the morphological analysis were 3.0 kV acceleration voltage, with a secondary electron detector, and 10 kV acceleration voltage, with a backscattered electron detector. In the chemical analysis by EDS, a 15 kV acceleration voltage was used. The samples were previously coated (sputtering) with gold palladium.

#### 2.4.6. Adsorption of Methylene Blue

For the determination of methylene blue adsorption on PLA and PLA-ZnO/TiO_2_ samples, an aqueous solution of 200 mg/L of methylene blue concentration was prepared. The adsorption experiments were carried out in 50 mL beakers with 20 mL of the solution and 0.02 g of polymeric nanocomposites, respectively. The beakers were placed on a stirring grill at room temperature and vigorously shaken at 600 rpm for 18 min. Every 2 min a sample was taken and placed in a vial for later reading in the UV-VIS spectrometer (Shimadzu UV-2401 PC) at a wavelength of 664 nm. All experiments were performed in triplicate.

The percentage of adsorption efficiency was calculated according to Equation (1):

(1) $$\%Adsorptionefficienty=\frac{C_{i}-C_{e}}{C_{i}}\times 100$$

where *C_i_* and *C_e_* are initial and final concentrations, respectively.

The adsorption capacity of the PLA and PLA ZnO/TiO_2_ samples was calculated with Equation (2) in equilibrium:

(2) $$q_{e}=\frac{\left(C_{i}-C_{e}\right)V}{m}$$

where *V* is the volume in L of solution and m is the amount of mass in mg of absorbent.

#### 2.4.7. Adsorption Isotherm

Langmuir's and Freundlich’s models can describe the adsorption equilibrium. To test both models, absorption isotherm data were fitted and the correlation coefficient (R^2^) was calculated using the trendline command in Microsoft Excel.

The Langmuir isotherm was calculated using the following Equation (3)

(3) $$\frac{C_{e}}{q_{e}}=\frac{C_{e}}{q_{m}}+\frac{1}{K_{Lqm}}$$

where *q_e_* (mg.g^−1^) and *C_e_* (mg.L^−1^) are the concentrations of the solid phase and liquid phase of the adsorbate in equilibrium, respectively; *q_m_* is the maximum adsorption capacity and *K_Lqm_* is the constant obtained from plotting *C_e_/Q_e_* versus *C_e_.*

The Freundlich isotherm was calculated using the following equation:

(4) $$Ln\left(q_{e}\right)=Ln\left(K_{F}\right)+\left(\frac{1}{n}\right)Ln\left(C_{e}\right)$$

where *K_F_* (mg.g^−1^) (L.mg^−1^) and 1/*n* are the Freundlich constants related to the adsorption capacity and *n* is the heterogeneity calculated with the lineal plot of *Ln(q_e_)* versus *Ln(C_e_)*.

#### 2.4.8. Desorption Studies

The desorption and regeneration of methylene blue adsorbents were studied during three successive cycles. In each cycle, the adsorbents were loaded with methylene blue by mixing 20 mg of the sample with 20 mL of the 200 mg/L dye concentration at room temperature. The mixture was stirred for 18 min. The methylene blue-loaded samples were separated by filtration. The percentage adsorption efficiency of the regenerated materials was determined by the same method mentioned above.

## 3. Results

### 3.1. Fourier-Transform Infrared Spectroscopy (FTIR)

**Figure 1:**
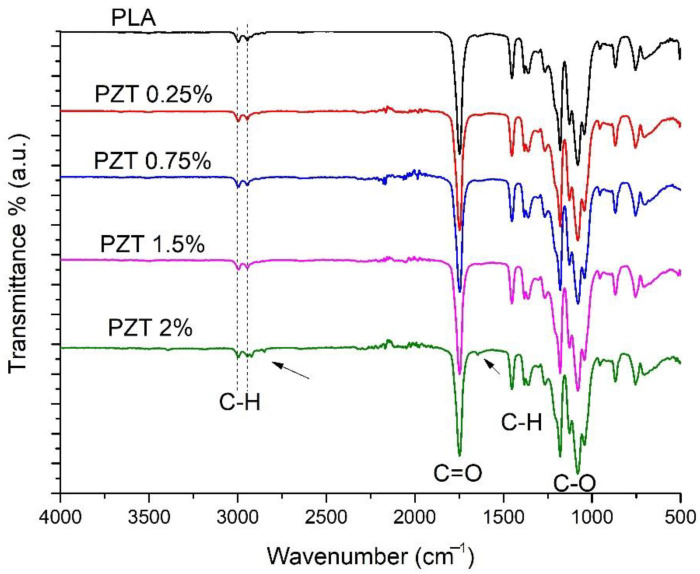
FTIR spectra of neat PLA and nanocomposite PZT at different concentrations.

Figure 1 shows the FTIR spectrum of pure PLA, where two peaks can be seen at 2997 and 2940 cm^−1,^ characteristic of the vibration of aliphatic C-H stretching [24]. A peak can also be seen at 1747 cm^−1,^ attributed to the stretching vibration of the carbonyl groups C=O of the ester group, the band that appears around 1453 cm^−1^ corresponding to the C-H bond symmetric deformation.

On the other hand, the appearance of the bands around 1262–1070 cm^−1^ is attributed to the C-O stretching vibration of the ether groups [25]. PZT nanocomposites present the same adsorption bands as pure PLA, as reported by Kim et al. for PLA-ZnO nanocomposites [26]. These authors indicate that there was no chemical interaction between PLA and ZnO. In this study, the PZT 2% sample presented two different bands of low intensity at 2850 cm^−1,^ assigned to the symmetric vibrations -CH_2_ and 1640 cm^−1^ corresponding to the hydroxyl group, both bands coming from the modification of the ZnO/TiO_2_ with lactic acid.

### 3.2. X-ray Diffraction (XRD)

**Figure 2:**
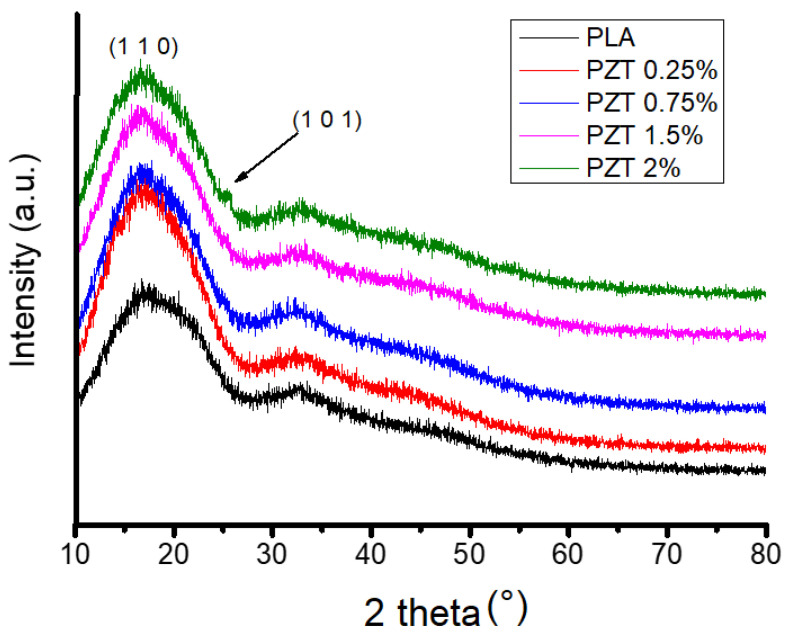
XRD spectra of neat PLA and nanocomposite PZT at different concentrations.

Figure 2 shows the XRD pattern of the PLA polymeric matrix and of the PLA-ZnO/TiO_2_ nanocomposites with different percentages of nanoparticles. In this figure, a broad intensity peak centered at 2θ = 17° ± 0.5 was observed in the pure PLA and the PZT nanocomposite, indicating that it is an amorphous structure as no crystalline peaks were observed. This agrees with what was reported by Pantani et al. [27], when they studied the PLA-ZnO nanocomposites. In addition, no change in the crystalline structure was observed when incorporating modified ZnO/TiO_2_ nanoparticles. Only an additional intensity peak was observed in the 2% PZT sample at 2θ = 25° because it was the sample with the highest load of nanoparticles. This was due to the presence of modified TiO_2_ nanoparticles as has already been previously reported in PLA-TiO_2_ nanocomposites [28]. Crystallinity results are as follows: 34 ± 1 for PLA and 50 ± 0.5, 52 ± 0.7, 53 ± 1 and 56 ± 1.2% for PZT, corresponding to 0.25, 0.75, 1.5 and 2%, respectively. It can be seen that the crystallinity index increases as the concentration of the nanoparticles increases. Several authors have reported that in the case of fillers, such as ZnO/TiO_2_ nanoparticles, they can act as crystallization nucleating agents [29,30,31].

### 3.3. Thermogravimetric Analysis (TGA)

**Figure 3:**
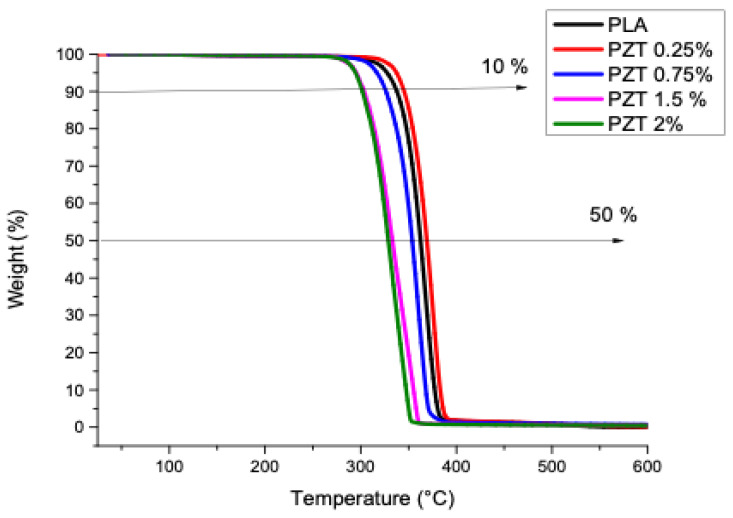
Thermogravimetric analysis of PLA and nanocomposites obtained.

The TGA analysis of the PLA and the polymeric compounds obtained is presented in Figure 3. These results show the onset of weight loss for the compounds and the PLA from 280 °C and stabilizing up to 388 °C. A comparison of the temperatures recorded at 10% and 50% weight loss was made (Table 2). It was observed that the only sample that increased its thermal stability was PZT 0.25%, having a temperature of 344 °C at 10% weight loss, which was higher compared to PLA (337 °C). Likewise, at 50% weight loss, the 0.25% PZT sample presented 368 °C, resulting in greater thermal stability than PLA (362 °C). This indicates that at this weight percentage there is a greater compatibility between the ZnO and TiO_2_ particles modified with lactic acid with the PLA polymeric matrix. Studies of PLA and nanoparticles such as modified nanoclays, through ultrasound-assisted extrusion, have shown that adding modified nanoparticles to some functional groups increases the thermal stability of polymeric nanocomposites [32].

The PZT 0.75%, PZT 1.5%, and PZT 2% samples presented lower temperature values at 10 and 50% weight loss compared to the polymer alone. This faster decomposition phenomenon is the reason for the evaporation of the carboxyl group, because the amount of functional groups that were present in the modified samples was around 43% [23].

For the obtained PLA, PZT 0.25%, PZT 0.75%, PZT 1% and PZT 2% samples, the amount of residue obtained at 580 °C was 0, 0.2, 0.5, 0.7 and 1.5%, respectively. Such results could be due to the amount of particles that remain in the extruder and to the carrying out of this method of extrusion assisted by ultrasound materials.

### 3.4. Differential Scanning Calorimetry (DSC)

**Figure 4:**
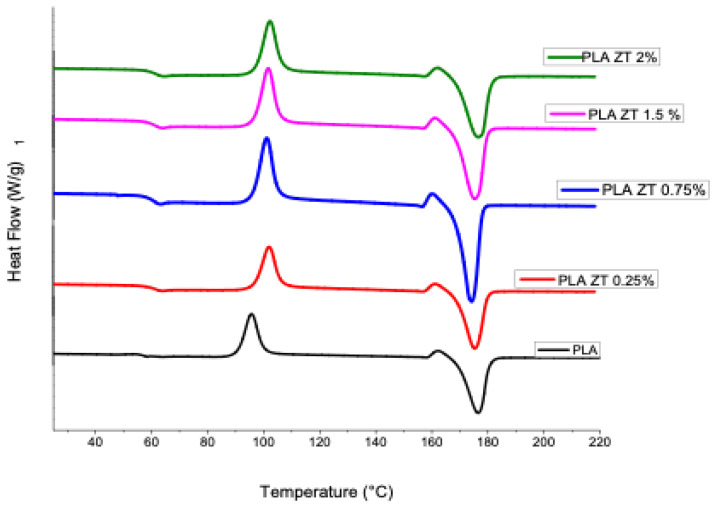
Differential scanning calorimetry of pure PLA and obtained compounds.

A DSC analysis was performed on the samples obtained (Figure 4). The values of melting temperatures, fusion energy and cold crystallization temperatures are presented in Table 3. The melting temperatures of the samples with modified nanoparticles were very similar to those obtained for PLA, with all the samples in the range of 174.30 to 176.6 °C, showing that there is no notable effect when adding these nanofillers to the polymeric matrix. In the cold crystallization temperatures (T_cc_), an increase is seen when increasing the content of modified ZnO and TiO_2_ nanoparticles, so that the pure PLA presented a T_cc_ of 98 °C and the sample with a higher content of PZT nanoparticles, 2%, a T_cc_ of 102.33 °C.

It must be taken into account that PLA has slow crystallization kinetics compared to other polymers, and it has been reported that the presence of nanoparticles in the polymeric matrix contributes to increasing the crystalline content of the materials [33].

Pure PLA showed a crystallization temperature (T_c_) of 96 °C and a melting temperature (T_m_) of 176 °C; the compounds did not show (T_c_). The increase in the cold crystallization temperature (T_cc_) with the addition of the nanoparticles implies that, during the cooling cycle, the higher particle content must have acted as a physical barrier to crystal growth [34].

### 3.5. Scanning Electron Microscopy (SEM)

**Figure 5:**
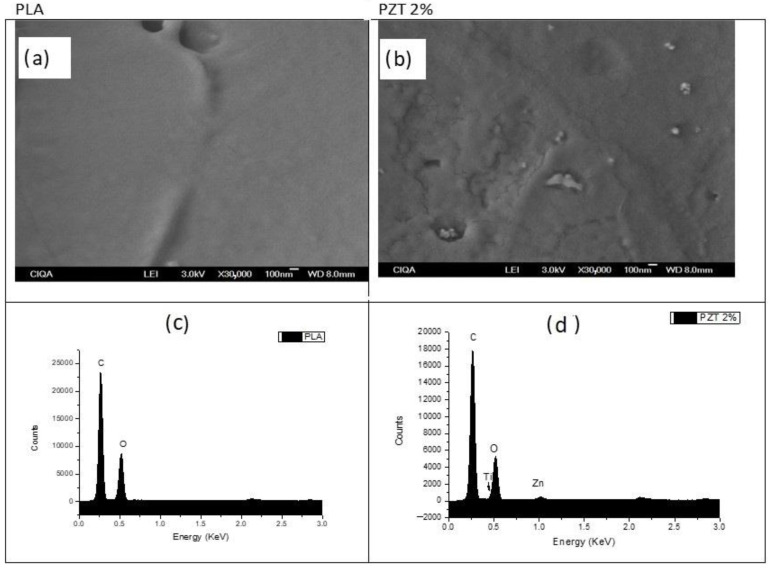
SEM images of (**a**) PLA and (**b**) 2% PZT samples at 30,000×. EDS analysis of (**c**) PLA and (**d**) 2% PZT samples.

Figure 5 presents the SEM images and the EDS (Energy Dispersive Spectroscopy) analysis of the PLA and of the sample with the highest percentage of modified TiO_2_ and ZnO nanoparticles (PZT 2%). Images were taken at a magnification of 30,000×. In the PLA polymer, only the surface of the polymer can be seen without any nanoparticles and some roughness on its surface. For the PZT 2% sample, some particles and agglomerates with different sizes can be seen at different magnifications, indicating that there was a good dispersion throughout the polymer of the two different particles having spherical morphology.

Maryam Azizi-Lalabadi et al. reported SEM images of nanocomposite films of PVA and TiO_2_/ZnO, obtained by mixing in their suspension, showing agglomerates of TiO_2_, ZnO and of the two particles together with different particle sizes. These films were mentioned as a good alternative for food packaging [35].

From the EDS analysis of the PLA polymer, only signals corresponding to the C (0.2 KeV) and O (0.52 KeV) atoms that make up said structurally pure polymer [36] were detected. The sample of PZT 2% with nanoparticles presented a concentration of C, O, Ti (0.46 KeV) and Zn (1.02 KeV) atoms, confirming the presence of these two nanoparticles in the polymeric nanocomposite.

### 3.6. Adsorption of Methylene blue

**Figure 6:**
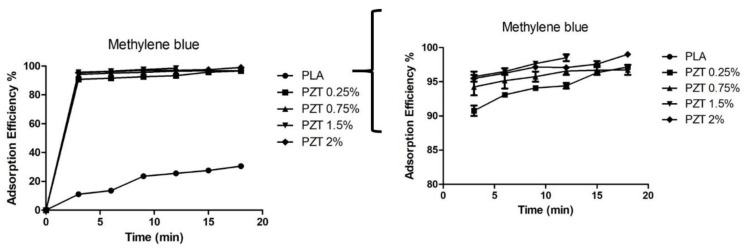
The adsorption efficiency of PLA and nanocomposite PZT at different concentrations (MB concentration = 200 mg/L; nanocomposite content = 20 mg/20 mL; T = 25 °C and t = 18 min). The second figure is the same, at a scale of 80 to 100% adsorption efficiency.

The adsorption efficiency of pure PLA and nanocomposites are shown in Figure 6. In pure PLA, it was observed that after 10 min adsorption equilibrium was reached with an adsorption efficiency of 25%. A 60% removal rate for cellulose/PLA/polyurethane membrane impregnated with cobalt nanoparticles has been reported [37]. In the PZT nanocomposites, it was observed that they had the same adsorption behavior in the first 3 min, and removal was close to 90% for all nanocomposites. This is due to the rapid uptake of MB molecules on the outer surface of the nanocomposite. The PZT 1.5% nanocomposite presented a 98.9% adsorption efficiency at 13 min. At this time the equilibrium of adsorption and desorption was reached for the elimination of dyes in wastewater. The contact time and adsorption are important. A shorter contact time allows efficient removal of contaminants when they are found in a treatment facility that receives large volumes of water [38]. The nanoparticles ZnO/TiO_2,_ unmodified and modified with lactic acid, showed 67 and 85% adsorption efficiency for methylene blue dyes in a time of 12 min, as reported in a previous study [23]. When these nanoparticles are incorporated as nanofillers in the PLA matrix, the percentage of adsorption efficiency increases.

Mohammad et al. studied the adsorption of PLLA/TiO_2_ nanofiber membranes for MB removal. They obtained 100% removal at a time of 60 min under light radiation. In this study, the nanocomposite at different concentrations of ZnO/TiO_2_ nanoparticles performed for a shorter time and with an adsorption efficiency greater than 95% [39].

The parameters and their predicted values by the Langmuir and Freundlich models are summarized in Table 4. The equilibrium data were adjusted in all the nanocomposites to the Langmuir isotherm with a correlation constant of R^2^ = 0.999. The adsorption process was homogeneous and adsorption sites formed a single layer. In addition, it was observed in the Langmuir isotherm data that the theoretical maximum adsorption (q_max_) agreed with the experimental data obtained, such as the percentage of removal. It was observed that the value of q_max_ is higher when it comes to nanocomposites with different charge concentrations. In a previous study of PLA nanocomposites with nanoclay, it was observed that they fit the same Langmuir model.

**Figure 7:**
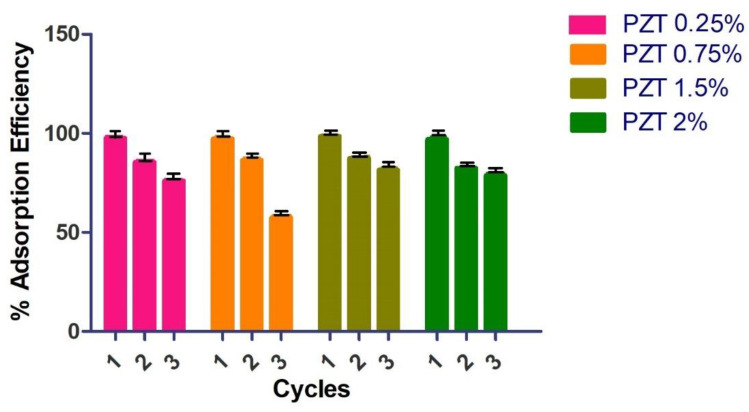
Effect of desorption cycles on methylene blue removal (MB concentration = 200 mg/L, nanocomposite PZT content = 20 mg/100 mL, T = 25 °C and 18 min).

To verify the regeneration and reuse of the nanocomposite, a process of adsorption and desorption of the material was carried out (Figure 7). The first time, the MB dye was adsorbed with a contact time of 18 min, and the material was filtered off and dried. The adsorbent nanocomposite was directly used for the following adsorption experiment. After three adsorption cycles, the PZT nanocomposite presented a good performance when reused. The adsorption efficiency percentages were greater than 80% except for the PZT 0.75% nanocomposite, which at the third cycle obtained a 60% MB adsorption efficiency. The nanocomposites can be collected quickly due to their particle size and do not require washing with solvents or acid solutions. The 1.5% PZT nanocomposite demonstrated rapid dye adsorption after each adsorption cycle.

Table 5 shows a comparison with other reported adsorbents. Having biodegradable materials, all the reported materials present a removal percentage greater than 90%.

However, the time is different for each of the materials, since they reach an equilibrium at a longer time. In the case of this study the time is 13 min, which suggests that the 1.5% PZT nanocomposite is a good candidate for the removal of dyes in aqueous solutions.

## 4. Discussion

The extrusion process consists of melting thermoplastic polymers, where a polymer nanocomposite can be obtained in a single step. The combination of thermal energy due to heating and the friction of the screws and cylinder allows the polymer pellets to melt, as well as to obtain a uniform mixing [43].

In recent years, this process has been accompanied by an ultrasound that is placed either at the end or in the intermediate zones, allowing a dispersion of the nanoparticles in the molten polymeric matrix [44]. This process is simple, solvent-free and easily scalable to an industrial level. For the formation of the nanocomposites, the ZnO/TiO_2_ nanoparticles are chemically modified with carboxylic groups. In a previous study by the authors, the lactic acid modification of these nanoparticles was chosen due to the 43% modification [23].

The effect of the ZnO/TiO_2_ nanoparticles and ultrasound can be observed in the spectroscopic characterization. For example, in the FTIR the characteristic signals of PLA were observed, and only in the PZT 2% sample were two bands of low intensity at 2850 cm^−1^, assigned to the symmetric vibrations -CH_2_ and 1640 cm^−1^ corresponding to the hydroxyl group, observed. The characteristic bands of ZnO and TiO_2_ were observed in the region in 400–700 cm^−1^. This agrees with what was reported by Buzarovska, where the PLA nanocomposite was studied with TiO_2_ nanoparticles functionalized with propanoic acid, where these bands were attributed to the chemical modification of the nanoparticles [45]. In the XRD analysis, only a slight change in the diffractogram was detected in the PZT 2% sample. The amorphous nature of PLA has also been observed [46]. The few changes observed in the XRD may be due to the weight percentage of the nanofiller and the poor chemical interaction that occurs between the matrix and the nanoparticles [47,48]. Crystallinity was calculated and it was observed that it increases when the concentration of the nanoparticles in the polymeric matrix increases.

In the thermal characterization of the PZT nanocomposites, the results (Figure 3) showed similar TGA curves, with no loss of water due to humidity observed. A significant mass loss was observed from 280 °C and stabilizing up to 388 °C, which corresponds to the PLA decomposition widely reported in the literature [48]. The mass that remained at the end corresponds to the inorganic material, that is, to the ZnO/TiO_2_ nanoparticles, which is very close to the amount of percentage by weight that was added. As seen in Figure 4, with the addition of ZnO/TiO_2_ nanoparticles, the cold crystallization temperature increased with the increase in the weight percentage of the nanoparticles. In contrast to pure PLA, the dispersion of the nanoparticles plays an important role in the behavior of crystallization [49].

The dispersion of the nanoparticles was studied by SEM. A few agglomerates and a homogeneous distribution of the nanoparticles were observed due to the ultrasound-assisted extrusion method that was used; modifying the nanoparticles helps to obtain a good dispersion and distribution. Elements C, O, Ti and Zn were found in the PZT sample 2%.

This nanocomposite was applied as an adsorbent material for dyes. Nowadays, the contamination of rivers in Mexico is a concern. Mainly, a solution is sought for the sanitation of the Atoyac River [50]. As has been reported by some of the authors, the problem is serious and the main contaminants present are dyes from textile industries.

In a previous study, the adsorption of dyes in nanoparticles alone was reported [23]. In this work, the nanocomposite with a biodegradable matrix was reported using these nanoparticles.

The adsorption mechanism of the PZT nanocomposite can occur through physisorption, chemisorption, or both. It can be carried out through electrostatic interaction between the OH- groups present in the modification of the nanoparticles and the S present in the methylene blue molecule, or in the carbonyl group of PLA. It can also be carried out through hydrogen bonds between the methylene blue molecule and the PZT nanocomposite. The particulate rough nanocomposite surface promotes physical adsorption sites.

## 5. Conclusions

This research contributes to the development of adsorbent materials using biodegradable nanocomposites such as PLA incorporating ZnO/TiO_2_ ceramic nanoparticles modified with renewable organic acids. The method for the preparation of the nanocomposite may be environmentally friendly since it does not use solvents or generate by-products. These new materials are an excellent alternative to building a filter based on non-woven fabrics, and thus help in water treatment, specifically the removal and degradation of dyes. The results of the characterization indicated by FTIR, the characteristic signals of PLA and the sample PZT 2% showed the bands corresponding to the carbon-hydrogen -CH_2_ bonds and the hydroxyl group, both coming from the modification of the ZnO/ TiO_2_ with lactic acid. By means of XRD, it was observed that when increasing the percentage of nanoparticle load, the crystallinity increases, due to the nature of the ceramic particles. In the SEM images, it was possible to observe that there was a good dispersion of the nanoparticles in the PLA polymeric matrix, and in adsorption the methylene blue dye revealed 98% dye adsorption in a time of 13 min for the 1.5% PZT sample. Additionally, this material could be used for 3 adsorption cycles.

In future, the use of PZT nanocomposites could be an alternative for removing dyes from wastewater. Nonetheless, more studies are needed to make this a reality and manufacture commercial non-woven fabrics that could help to eliminate these types of pollutants. These studies would include adsorption in a continuous system as well as determining the effect of pH and temperature. Additionally, it would be worth studying the adsorption of inorganic pollutants and emerging pollutants. This knowledge would help to find an alternative solution with a comprehensive approach.

## Figures and Tables

**Table 1:** Identification of the PLA-ZnO/TiO_2_ nanocomposites with different percentages of additives (0, 0.25, 0.75 and 2 wt.%).

Sample Code	Description
PLA	Pristine PLA
PZT 0.25%	PLA with 0.25% of ZnO/TiO_2_ modified with lactic acid
PZT 0.75%	PLA with 0.75% of ZnO/TiO_2_ modified with lactic acid
PZT 1.5%	PLA with 1.5% of ZnO/TiO_2_ modified with lactic acid
PZT 2%	PLA with 2% of ZnO/TiO_2_ modified with lactic acid

**Table 2:** Thermal properties of the samples obtained.

Sample	Temperature at Different Level of Weight Loss		Amount of Residue at 580 °C
	T_10%_ (°C)	T_50%_ (°C)	(%)
PLA	337	362	0.00
PZT 0.25%	344	368	0.21
PZT 0.75%	325	354	0.5
PZT 1.5%	304	333	0.7
PZT 2%	300	328	1.00

**Table 3:** Differential scanning calorimetry (DSC) data for PLA and nanocomposites obtained.

Sample	Melting Temperature (°C) T_m_	Melting Enthalpy (J/g)	Cold Crystallization Temperature (°C) T_cc_	Cold Crystallization Entalphy (J/g) (T_cc_)
PLA	176.1	58.28	98.0	30.97
PZT 0.25%	175.4	48.73	102.0	33.25
PZT 0.75%	174.3	45.95	101.1	36.10
PZT 1.5%	175.8	47.70	101.6	34.95
PZT 2%	176.6	40.18	102.3	30.31

**Table 4:** Parameters of the isotherm constants and correlation coefficients were calculated for MB adsorption in PZT nanocomposites.

Sample	Langmuir			Freundlich		
	k	q_max_	R^2^	n	K_F_	R^2^
PLA	0.02	108	0.9876	3.1	9.87	0.8234
PZT 0.25%	0.011	222	0.9995	2.2	120	0.9048
PZT 0.75%	0.010	207	0.9998	5.8	308	0.9927
PZT 1.5%	0.010	203	0.9999	6.3	337	0.9986
PZT 2%	0.013	206	0.9999	5.9	315	0.9979

**Table 5:** A comparison between the performance of the polymeric adsorbents in previous works and nanocomposite PZT in this study for the removal of methylene blue.

Adsorbent Type	% Remove	Time Contact	Ref.
Boehmite@Fe_3_O_4_@PLA@SiO_2_	95	180 min	[40]
PLLA/TiO_2_	100	60 min	[39]
PLA/C20A Nanoclay	97	60 min	[32]
PAni-TiO_2_	87	60 min	[41]
ZnO-Chitosan	99.95	160 min	[42]
PZT 1.5%	98.9	13 min	This study

## Data Availability

Not applicable.

## References

[1] Gryta M. (2011). Water desalination by membrane distillation. Desalination, Trends and Technologies.

[2] Velusamy S., Roy A., Sundaram S., Kumar Mallick T. (2021). A review on heavy metal ions and containing dyes removal through graphene oxide-based adsorption strategies for textile wastewater treatment. Chem. Rec..

[3] Sonune A., Ghate R. (2004). Developments in wastewater treatment methods. Desalination.

[4] Bagastyo A.Y., Keller J., Poussade Y., Batstone D.J. (2011). Characterisation and removal of recalcitrants in reverse osmosis concentrates from water reclamation plants. Water Res..

[5] Wang H., Mi X., Li Y., Zhan S. (2020). 3D graphene-based macrostructures for water treatment. Adv. Mater..

[6] Heemskerk S., Van Haren F.M., Foudraine N.A., Peters W.H., Van Der Hoeven J.G., Russel F.G., Masereeuw R., Pickkers P. (2008). Short-term beneficial effects of methylene blue on kidney damage in septic shock patients. Intensive Care Med..

[7] Cabello-Alvarado C.J., Quiñones-Jurado Z.V., Cruz-Delgado V.J., Avila-Orta C.A. (2020). Pigmentation and degradative activity of TiO_2_ on polyethylene films us-ing Masterbatches fabricated using variable-frequency ultrasound-assisted melt-extrusion. Materials.

[8] Li Y.F., Aschauer U., Chen J., Selloni A. (2014). Adsorption and reactions of O_2_ on anatase TiO_2_. Acc. Chem. Res..

[9] Gan W., Shang X., Li X.H., Zhang J., Fu X. (2019). Achieving high adsorption capacity and ultrafast removal of methylene blue and Pb2+ by graphene-like TiO_2_@ C. Colloids Surf. A Physicochem. Eng. Asp..

[10] Prasad K., Jha A.K. (2009). ZnO nanoparticles: Synthesis and adsorption study. Nat. Sci..

[11] Chantes P., Jarusutthirak C., Danwittayakul S. A Comparison Study of Photocatalytic Activity of TiO_2_ and ZnO on the Degradation of Real Batik Wastewater. Proceedings of the International Conference on Biological, Environment and Food Engineering (BE-FE-2015).

[12] Zhang F., Lan J., Yang Y., Wei T., Tan R., Song W. (2013). Adsorption behavior and mechanism of methyl blue on zinc oxide nanoparticles. J. Nanopart. Res..

[13] Cabello C., Rincón S., Bartolo P., Ruiz-Espinoza J., Zepeda A. (2018). Incorporation of organic groups on the surface of multi-walled carbon nanotubes using an ultrasonic tip. Fuller. Nanotub. Carbon Nanostruct..

[14] Rupa E., Kaliraj L., Abid S., Yang D., Jung S. (2019). Synthesis of a Zinc Oxide Nanoflower Photocatalyst from Sea Buckthorn Fruit for Degradation of Industrial Dyes in Wastewater Treatment. Nanomaterials.

[15] Dodoo-Arhin D., Asiedu T., Agyei-Tuffour B., Nyankson E., Obada D., Mwabora J.M. (2021). Photocatalytic degradation of Rhodamine dyes using zinc oxide nanoparticles. Mater. Today Proc..

[16] Hu J., Wu D., Feng Q., Wei A., Song B. (2020). Soft High-Loading TiO_2_ Composite Biomaterial Film as an Efficient and Recyclable Catalyst for Removing Methylene Blue. Fibers Polym..

[17] Rajagopal S., Paramasivam B., Muniyasamy K. (2020). Photocatalytic removal of cationic and anionic dyes in the textile wastewater by H2O2 assisted TiO_2_ and micro-cellulose composites. Sep. Purif. Technol..

[18] Xu Q., Ju Y., Ge H. (2012). Kinetics of TiO_2_/Polyurethane Films for Degradation of Organic Pollutants in Water. Adv. Mater. Res..

[19] Shen L., Huang Z., Liu Y., Li R., Xu Y., Jakaj G., Lin H. (2020). Polymeric Membranes Incorporated with ZnO Nanoparticles for Membrane Fouling Mitigation: A Brief Review. Front. Chem..

[20] Vatanpour V., Nekouhi G., Esmaeili M. (2020). Preparation, characterization and performance evaluation of ZnO deposited polyethylene ultrafiltration membranes for dye and protein separation. J. Taiwan Inst. Chem. Eng..

[21] Dugan J.S. (2001). Novel properties of PLA fibers. Int. Nonwovens J..

[22] Mittal V. (2011). Nanocomposites with Biodegradable Polymers: Synthesis, Properties, and Future Perspectives.

[23] Andrade-Guel M., Cabello-Alvarado C., Bartolo-Pérez P., Medellin-Banda D.I., Ávila-Orta C.A., Cruz-Ortiz B., Pliego G.C. (2022). Surface modification of TiO_2_/ZnO nanoparticles by organic acids with enhanced methylene blue and rhodamine B dye adsorption properties. RSC Adv..

[24] Cuiffo M.A., Snyder J., Elliott A.M., Romero N., Kannan S., Halada G.P. (2017). Impact of the fused deposition (FDM) printing process on polylactic acid (PLA) chemistry and structure. Appl. Sci..

[25] Wang X.J., Huang Z., Wei M.Y., Lu T., Nong D.D., Zhao J.X., Teng L.J. (2019). Catalytic effect of nanosized ZnO and TiO_2_ on thermal degradation of poly (lactic acid) and isoconversional kinetic analysis. Thermochim. Acta.

[26] Kim I., Viswanathan K., Kasi G., Sadeghi K., Thanakkasaranee S., Seo J. (2019). Poly (lactic acid)/ZnO bionanocomposite films with positively charged ZnO as potential antimicrobial food packaging materials. Polymers.

[27] Pantani R., Gorrasi G., Vigliotta G., Murariu M., Dubois P. (2013). PLA-ZnO nanocomposite films: Water vapor barrier properties and specific end-use characteristics. Eur. Polym. J..

[28] Deghiche A., Haddaoui N., Zerriouh A., Fenni S.E., Cavallo D., Erto A., Benguerba Y. (2021). Effect of the stearic acid-modified TiO_2_ on PLA nanocomposites: Morphological and thermal properties at the microscopic scale. J. Environ. Chem. Eng..

[29] Picard E., Espuche E., Fulchiron R. (2011). Effect of an organo-modified montmorillonite on PLA crystallization and gas barrier properties. Appl. Clay Sci..

[30] Črešnar K.P., Zemljič L.F., Papadopoulos L., Terzopoulou Z., Zamboulis A., Klonos P.A., Pissis P. (2021). Effects of Ag, ZnO and TiO_2_ nanoparticles at low contents on the crystallization, semicrystalline morphology, interfacial phenomena and segmental dynamics of PLA. Mater. Today Commun..

[31] Kaseem M., Hamad K., Ur Rehman Z. (2019). Review of recent advances in polylactic acid/TiO_2_ composites. Materials.

[32] Andrade-Guel M., Cabello-Alvarado C., Romero-Huitzil R.L., Rodríguez-Fernández O.S., Ávila-Orta C.A., Cadenas-Pliego G., Cepeda-Garza J. (2021). Nanocomposite PLA/C20A Nanoclay by Ultrasound-Assisted Melt Extrusion for Adsorption of Uremic Toxins and Methylene Blue Dye. Nanomaterials.

[33] Ortenzi M.A., Basilissi L., Farina H., Di Silvestro G., Piergiovanni L., Mascheroni E. (2015). Evaluation of crystallinity and gas barrier properties of films obtained from PLA nanocomposites synthesized via “in situ” polymerization of l-lactide with silane-modified nanosilica and montmorillonite. Eur. Polym. J..

[34] Ojijo V., Sinha Ray S., Sadiku R. (2012). Effect of nanoclay loading on the thermal and mechanical properties of biodegradable polylactide/poly [(butylene succinate)-co-adipate] blend composites. ACS Appl. Mater. Interfaces.

[35] Azizi-Lalabadi M., Alizadeh-Sani M., Divband B., Ehsani A., McClements D.J. (2020). Nanocomposite films consisting of functional nanoparticles (TiO_2_ and ZnO) embedded in 4A-Zeolite and mixed polymer matrices (gelatin and polyvinyl alcohol). Food Res. Int..

[36] Fukushima K., Kimura Y. (2006). Stereocomplexed polylactides (Neo-PLA) as high-performance bio-based polymers: Their formation, properties, and application. Polym. Int..

[37] El-Sayed M.M., Elsayed R.E., Attia A., Farghal H.H., Azzam R.A., Madkour T.M. (2021). Novel nanoporous membranes of bio-based cellulose acetate, poly (lactic acid) and biodegradable polyurethane in-situ impregnated with catalytic cobalt nanoparticles for the removal of Methylene Blue and Congo Red dyes from wastewater. Carbohydr. Polym. Technol. Appl..

[38] Otero M., Rozada F., Calvo L.F., Garcıa A.I., Moran A. (2003). Elimination of organic water pollutants using adsorbents obtained from sewage sludge. Dye. Pigment..

[39] Mohammad N., Atassi Y. (2021). TiO_2_/PLLA electrospun nanofibers membranes for efficient removal of methylene blue using sunlight. J. Polym. Environ..

[40] Alinezhad H., Zabihi M., Kahfroushan D. (2020). Design and fabrication the novel polymeric magnetic boehmite nanocomposite (boehmite@Fe_3_O_4_@PLA@SiO_2_) for the remarkable competitive adsorption of methylene blue and mercury ions. J. Phys. Chem. Solids.

[41] Ahmad R., Mondal P.K. (2012). Adsorption and photodegradation of methylene blue by using PAni/TiO_2_ nanocomposite. J. Dispers. Sci. Technol..

[42] Zango Z.U., Dennis J.O., Aljameel A.I., Usman F., Ali M.K.M., Abdulkadir B.A., Ibnaouf K.H. (2022). Effective Removal of Methylene Blue from Simulated Wastewater Using ZnO-Chitosan Nanocomposites: Optimization, Kinetics, and Isotherm Studies. Molecules.

[43] Jamshidian M., Tehrany E.A., Imran M., Jacquot M., Desobry S. (2010). Poly-lactic acid: Production, applications, nanocomposites, and release studies. Compr. Rev. Food Sci. Food Saf..

[44] Isayev A.I., Kumar R., Lewis T.M. (2009). Ultrasound assisted twin screw extrusion of polymer–nanocomposites containing carbon nanotubes. Polymer.

[45] Buzarovska A. (2013). PLA nanocomposites with functionalized TiO_2_ nanoparticles. Polym. Plast. Technol. Eng..

[46] Mallick S., Ahmad Z., Touati F., Bhadra J., Shakoor R.A., Al-Thani N.J. (2018). PLA-TiO_2_ nanocomposites: Thermal, morphological, structural, and humidity sensing properties. Ceram. Int..

[47] Segura González E.A., Olmos D., Lorente M.Á., Vélaz I., González-Benito J. (2018). Preparation and characterization of polymer composite materials based on PLA/TiO_2_ for antibacterial packaging. Polymers.

[48] Wu D., Wu L., Wu L., Xu B.I.N., Zhang Y., Zhang M. (2007). Nonisothermal cold crystallization behavior and kinetics of polylactide/clay nanocomposites. J. Polym. Sci. Part B Polym. Phys..

[49] Ogata N., Jimenez G., Kawai H., Ogihara T. (1997). Structure and thermal/mechanical properties of poly (l-lactide)-clay blend. J. Polym. Sci. Part B Polym. Phys..

[50] Ávila Orta C.A., de Lourdes Hernández-Rodríguez M., Lozano Morales S.A. Río Atoyac: Hacia Una Gestión Integral de una Problemática Multifactorial. https://agua.org.mx/biblioteca/rio-atoyac-hacia-una-gestion-integral-de-una-problematica-multifactorial/.

